# CT texture features and lung shunt fraction measured using ^99m^Tc-macroaggregated albumin SPECT/CT before trans-arterial radioembolization for hepatocellular carcinoma patients

**DOI:** 10.1038/s41598-023-49787-7

**Published:** 2023-12-15

**Authors:** Jae Hwan Lee, Chong-ho Lee, Minuk Kim, Yoo Sung Song, Chang Jin Yoon, Won Woo Lee

**Affiliations:** 1 Department of Radiology, Seoul National University Bundang Hospital, Bundang-gu, Seongnam-si, Gyeonggi-do Republic of Korea; 2 Department of Radiology, Seoul National University College of Medicine, Jongno-gu, Seoul, Republic of Korea; 3 Institute of Radiation Medicine, Seoul National University Medical Research Center, Jongno-gu, Seoul, Republic of Korea; 4 Department of Radiology, Seoul Metropolitan Government-Seoul National University Boramae Medical Center, Seoul, Korea; 5 Department of Nuclear Medicine, Seoul National University Bundang Hospital, Bundang-gu, Seongnam-si, Gyeonggi-do Republic of Korea; 6 Department of Nuclear Medicine, Seoul National University College of Medicine, Seoul, Republic of Korea; 7 Department of Health Science and Technology, The Graduate School of Convergence Science and Technology, Seoul National University, Suwon-si, Gyeonggi-do Republic of Korea

**Keywords:** Hepatocellular carcinoma, Cancer imaging

## Abstract

The aim of this study is to determine whether contrast-enhanced computed tomography (CECT)-based texture parameters can predict high (> 30 Gy) expected lung dose (ELD) calculated using ^99m^Tc macroaggregated albumin single-photon emission computed tomography/computed tomography (SPECT/CT) for pre-trans-arterial radioembolization (TARE) dosimetry. 35 patients were analyzed, with a treatable planned dose of ≥ 200 Gy for unresectable hepatocellular carcinoma (HCC). Lung shunt fraction (LSF) was obtained from planar and SPECT/CT scans. Texture features of the tumor lesion on CECT before TARE were analyzed. Univariate and multivariate linear regression analyses were performed to determine potential ELD > 30 Gy predictors. Among the 35 patients, nine (25.7%) had ELD > 30 Gy, and had a higher LSF than the ELD ≤ 30 Gy group using the planar (20.7 ± 8.0% vs. 6.3 ± 3.3%; *P* < 0.001) and SPECT/CT (12.4 ± 5.1% vs. 3.5 ± 2.0%; *P* < 0.001) scans. The tumor integral total (HU × L) value was a predictor for high LSF using SPECT/CT, with an area under the curve, sensitivity, and specificity of 0.983 (95% confidence interval: 0.869–1.000, *P* < 0.001), 100%, and 88.5%, respectively. The tumor integral total value is an imaging marker for predicting ELD > 30 Gy. Applying CECT texture analysis may assist in reducing time and cost in patient selection and modifying TARE treatment plans.

## Introduction

Yttrium-90 (^90^Y) trans-arterial radioembolization (TARE) is increasingly being used to treat hepatocellular carcinoma (HCC)^1^. The recent Barcelona Clinic Liver Cancer prognosis and treatment strategy guideline posit TARE as a palliative option for the intermediate stage and a curative option for early stage (0 and A) lesions (single lesion with < 8 cm)^2^.

In practice, TARE is preceded by planning angiography and ^99m^Tc-macroaggregated albumin (MAA) scans^3^. The pre-TARE ^99m^Tc-MAA scans aim to calculate the effective target dose of ^90^Y for lesions, extrahepatic perfusion, and lung shunt fraction (LSF)^4^. TARE is contraindicated for glass microspheres if the expected lung dose is > 30 Gy, and for resin microspheres if expected LSF is > 20% ^5–7^. However, recent studies have reported better outcomes with a higher target dose of up to 400 Gy to the tumor^3,8–11^. Moreover, a high infusion ^90^Y dose can represent a double-edged sword by improving tumor response or increasing the chance of non-target radiation injury, such as fatal radiation pneumonitis. Therefore, accurate estimation of lung dose and appropriate adjustment of ^90^Y dose before TARE is mandatory.

A few studies have reported imaging findings as potential predictors for LSF, such as dysmorphic intra-tumoral vessels, early visualization of the hepatic vein, macrovascular invasion, substantial tumor burden, and infiltrative tumor^12–14^. Nonetheless, there is still limited amount of data available regarding its clinical utility in predicting LSF.

 Standard protocols require angiography and ^99m^Tc-MAA scan 1–2 weeks before TARE treatment to plan for tumor dose and exclude unsuitable candidates with aberrant vessel anatomy and high lung shunt. Accurate data on the drop-out rate with highly predicted lung shunt or normal liver dosages on ^99m^Tc-MAA scans is lacking; however, previous studies have reported a drop-out rate of 10–20%^3,9,15^. The patients who are dropped out after ^99m^Tc-MAA scans unavoidably experience a time delay until the decision of the next treatment option. Therefore, reducing the number of patients in this category would be clearly beneficial. Since contrast-enhanced computed tomography (CECT) is also performed for diagnostic purposes before TARE to estimate tumor and total liver volume for dosimetry calculation, we sought to determine how CT scans could assist in predicting more suitable candidates for TARE therapy. In this study, we analyzed the textural features of CECT of patients with HCC and correlated them with the LSF measured with single-photon emission computed tomography/computed tomography (SPECT/CT) who were referred for pre-TARE dosimetry. Texture analysis of CT has several merits in terms of objective, quantitative assessments compared with morphological CT analysis. SPECT/CT also has its merits in terms of accurate quantitative assessment compared with planar gamma scans. We hypothesize that certain parameters of the CECT are associated with high LSF on ^99m^Tc-MAA SPECT/CT and thus can aid in patient selection for TARE.

## Methods

### Patients

This retrospective study was approved by an institutional review board (IRB) of Seoul National University Bundang Hospital, and the need for informed consent was waived by the same IRB (IRB no. B-2201-734-104). All methods were performed in accordance with the relevant guidelines and regulations. An electronic medical record was searched from May 2018 to June 2022 to identify patients referred for TARE for HCC. There were 42 patients in the initial database. Among them, three patients with a history of trans-arterial chemoembolization and four without pre-treatment dynamic CECT imaging were excluded. Finally, 35 patients (male:female = 28:7, age: 66.9 ± 10.7 years) were included in the analysis.

### CECT protocol

Dynamic three- or four-phase CECT imaging was performed before planning angiography with scanners from multiple vendors in our institution using the following parameters: tube current of 150–250 mAs, peak tube voltage of 100–120 kVp, slice thickness of 3–5 mm, and a reconstruction interval of 2.0–3.0 mm^6,16^. Briefly, precontrast, hepatic arterial, portal venous, and delayed phase images were obtained under bolus tracking following the institution’s liver CT protocols^12^.

### Planning angiography

All patients underwent celiac angiography and cone-beam CT-common hepatic arteriography to evaluate anatomic information and tumor feeders. ^99m^Tc-MAA was injected at the sectional or lobar hepatic artery, which supplied the target tumor.

### ^99m^Tc-MAA imaging

^99m^Tc-MAA planar and SPECT/CT images were acquired within 2 h after injecting 74–185 MBq of ^99m^Tc-MAA into selected hepatic arteries. Images were acquired with NM/CT 670 and NM/CT 670 Pro cameras (GE Healthcare, Illinois, USA). First, anterior and posterior planar images were acquired, followed by SPECT/CT images. Lung and liver SPECT images were acquired under the following conditions: low-energy high-resolution collimator, 140 keV energy peak with a window of ± 10% (126–154 keV), step and shoot mode (5 s/step, 3° angle, total 120 steps) ^17^. Lung and liver CT images were also acquired under the following conditions: tube voltage 120 KVp and 30 mA current. The SPECT/CT images were reconstructed using the ordered subset expectation maximization (OSEM) method (two iterations, ten subsets), with CT attenuation correction, scatter correction for liver images, resolution recovery, and Butterworth filter application (frequency 0.48, order 10).

### ^99m^Tc-MAA SPECT/CT image analysis

Image analysis was performed using dedicated software (Xeleris 4DR, GE Healthcare, Illinois, USA). Two compartments were analyzed for ^99m^Tc-MAA planar scans, including the lungs and whole liver. Regions of interest (ROIs) for the lungs and whole liver were drawn on the anterior and posterior images. LSF was acquired using equation (Eq. 1), using the geometric mean count of anterior and posterior ROIs.

1 $$\mathrm{Lung \;shunt \;fraction} = 100 \times \frac{\mathrm{Lung \;count}}{\mathrm{Lung \;count }+\mathrm{ Whole \;liver \;count}}$$

Three compartments were analyzed for ^99m^Tc-MAA SPECT/CT scans, including the lung, tumor, and normal liver. The normal liver denotes the compartment of the entire liver, with the exclusion of the tumor. Total counts of each compartment were acquired. Volume of interests (VOIs) of each compartment were generated by manually drawing ROIs on every image slice, followed by volume rendering^15^. LSF for ^99m^Tc-MAA SPECT/CT was acquired using Eq. (2).

2 $$\mathrm{Lung \;shunt \;fraction }= 100 \times \frac{Lung \;count}{Lung \;count + Tumor \;count + Normal \;liver \;count}$$

The planned injection activity of TARE was calculated using Eq. (3)^18^ to target the absorbed dose to the tumor at 200 Gy^3,8,19^, based on the multi-compartment MIRD method ^20,21^. The absorbed dose of the lung and normal liver was also calculated using Eq. (3), with the planned injection activity being inputted to estimate the target tumor absorbed dose of 200 Gy. Patients were considered unsuitable for TARE therapy if the expected absorbed dose exceeded 30 Gy for the lung or the normal liver^22^.

3 $$\mathrm{Absorbed \;dose }({\text{Gy}}) = \frac{\left. 49.67 \;[\mathrm{Initial \;radioactivity \;in \;the \;compartment }({\text{Gbq}})\right]}{\left.Mass (Kg\right)}$$

The volume of the total lungs was acquired on the ^99m^Tc-MAA SPECT/CT scans, whereas the volumes of the tumor and normal liver were obtained on the liver CT scans. Volumes were measured from VOIs generated by drawing ROIs on every axial slice. Lung volumes were acquired with Xeleris 4DR (GE Healthcare), and tumor and liver volumes were acquired with a dedicated software (MIM Maestro, version 6.7, Ohio, USA). Tissue density was assumed to be 1.03 kg/L for the liver and tumor and 0.3 kg/L for the lungs^4,23^. The tumor-to-normal liver ratio (TNR) of the uptakes was also calculated ^24^.

4 $$\mathrm{TNR }= \frac{AT\left[MBq\right]/MT\left[Kg\right]}{ANL\left[MBq\right]/MNL\left[Kg\right]}$$

*A* and *M* indicate the activity and mass of the tumor (*T*) and normal liver (*NL*) compartments.

### CECT texture analysis

Two radiologists (J.H.L and C.L) and one nuclear medical doctor analyzed CECT images in consensus using MIM Maestro. The tumor VOIs were generated by manually drawing ROIs on every arterial phase axial image, and the following parameters of the tumor lesion were acquired; integral total Hounsfield unit × L (HU × L) value, kurtosis (–), maximum HU, mean HU, median HU, minimum HU, skewness (–), standard deviation of HU, and total HU. The integral total (HU × L) value within the tumor VOI was automatically calculated and defined as the sum of all the intensities within the contour divided by the number of voxels within the contour multiplied by the contour volume.

### Statistics

Continuous and categorical variables were compared using the Mann–Whitney U and chi-square test. Univariate linear regression was performed to determine the predictive factors for expected lung dose (ELD) > 30 Gy. Among significant factors with a variance inflation factor (VIF) higher than 10, only the factor with the highest VIF value was included in the multivariate linear regression analysis^25^. Receiver operating characteristic (ROC) curve analysis for predicting patients with ELD > 30 Gy was done. We performed 1000 bootstrap replications, each involving resampling with replacement and fitting the regression model to these samples. The statistical analysis was conducted using commercial software packages (MedCalc, version 20.023, MedCalc Software, Belgium, and SPSS Statistics, version 26, IBM, United States). The statistical significance was set at *P* values less than 0.05.

## Results

### Patients

35 patients with unresectable HCC referred for TARE were analyzed. Table 1 summarizes the demographics of patients.

**Table 1:** Demographics of the patients included in the study.

Variable	
Age	66.9 ± 10.7 (years)
Sex	
Male, number (%)	28 (80.0)
Women, number (%)	7 (20.0)
Etiology	
HBV, number (%)	17 (48.6)
Alcohol, number (%)	7 (20.0)
Others, number (%)	6 (17.1)
NASH, number (%)	4 (11.4)
HCV, number (%)	1 (2.8)
Distribution	
Solitary, number (%)	28 (80.0)
Multifocal, number (%)	7 (20.0)
Mean size, cm	10.2 ± 4.52
Child–Pugh class	
Class A, number (%)	35 (100.0)
Mean LSF, %	
Using planar scan	9.8 ± 8.1
Using SPECT/CT	5.6 ± 5.0

*HBV *Hepatitis B virus,* NASH *nonalcoholic steatohepatitis,* HCV *Hepatitis C virus,* LSF *lung shunt fraction.

### ^99m^Tc-MAA SPECT/CT image analysis

The average time interval between the ^99m^Tc-MAA injection and the start of ^99m^Tc-MAA SPECT/CT scan acquisition was 65.8 ± 44.7 min. LSF values obtained from planar scans were higher than those obtained from SPECT/CT (10.1 ± 8.1% vs 5.8 ± 5.0%, *P* < 0.001). The average tumor uptake of ^99m^Tc-MAA was 73.7 ± 17.8%, whereas the average normal liver uptake of ^99m^Tc-MAA was 20.5 ± 18.8% from SPECT/CT. The average TNR was 43.4 ± 58.6, measured on ^99m^Tc-MAA SPECT/CT images. On ^99m^Tc-MAA SPECT/CT analysis with an estimated target tumor dose of 200 Gy, nine patients (25.7%) were expected to receive ELD > 30 Gy, whereas four (11.4%) were expected to receive normal liver doses > 30 Gy (Table 2). Two were expected to receive both ELD and liver doses of > 30 Gy. The value of LSF measured by planar scan and by SPECT/CT were significantly higher in the patient group with ELD > 30 Gy compared to the patient group with ELD ≤ 30 Gy (*P* < 0.001, respectively). The value of normal liver uptake using SPECT/CT was significantly lower in the patient group with an ELD > 30 Gy (*P* = 0.04) compared to the patient group with ELD ≤ 30 Gy. Between the patient group with an expected normal liver dose of ≤ 30 Gy and those with an expected normal liver dose of > 30 Gy, TNR was significantly higher in the patient group with an expected normal liver dose of ≤ 30 Gy (*P* < 0.01).

**Table 2:** Comparison of ^99m^Tc-MAA imaging parameters between the ELD ≤ 30 Gy and ELD > 30 Gy groups, and between the expected liver dose ≤ 30 Gy and expected liver dose > 30 Gy groups.

	ELD ≤ 30 Gy (n = 26)	ELD > 30 Gy (n = 9)	P value
Planar LSF (%)	6.3 ± 3.3	20.7 ± 8.0	< 0.001
SPECT/CT LSF (%)	3.5 ± 2.0	12.4 ± 5.1	< 0.001
SPECT/CT tumor uptake (%)	71.6 ± 19.5	79.7 ± 10.4	0.47
SPECT/CT normal liver uptake (%)	24.9 ± 19.3	7.8 ± 9.3	0.04
TNR	52.0 ± 65.7	18.4 ± 13.9	0.10

	Expected liver dose ≤ 30 Gy (n = 31)	Expected liver dose > 30 Gy (n = 4)	P value
Planar LSF (%)	10.3 ± 8.6	8.6 ± 1.3	0.63
SPECT/CT LSF (%)	5.6 ± 5.2	7.3 ± 3.5	0.19
SPECT/CT tumor uptake (%)	75.2 ± 17.2	61.9 ± 20.7	0.21
SPECT/CT normal liver uptake (%)	19.2 ± 18.7	30.8 ± 18.7	0.23
TNR	48.5 ± 60.5	4.1 ± 1.3	< 0.01

*ELD* expected lung dose, *LSF* lung shunt fraction, *TNR* tumor-to-normal liver ratio.

### Analysis of CECT texture parameters

Table 3 summarizes the CECT texture parameters for the patient group with ELD ≤ 30 Gy and ELD > 30 Gy. The patient group with ELD > 30 Gy had higher tumor volume, total liver volume, tumor-to-liver volume ratio, tumor integral total (HU × L) value, and total tumor HU value compared with the ELD ≤ 30 Gy group (*P* < 0.001, respectively). However, the ELD ≤ 30 Gy group had a higher mean HU value of the tumor than the ELD > 30 Gy group (*P* = 0.04).

**Table 3:** Comparison of parameters from CECT texture analysis between the ELD ≤ 30 Gy and ELD > 30 Gy groups.

	ELD ≤ 30 Gy (n = 26)	ELD > 30 Gy (n = 9)	P value
Lung volume (L)	2.82 ± 0.69	2.47 ± 0.53	0.18
Tumor volume (L)	0.24 ± 0.21	1.50 ± 0.77	< 0.001
Total liver volume (L)	1.48 ± 0.43	2.77 ± 0.76	< 0.001
Normal liver volume (L)	1.24 ± 0.39	1.27 ± 0.20	0.47
Tumor-to-liver volume ratio	0.15 ± 0.12	0.51 ± 0.13	< 0.001
Tumor integral total (HU × L) value	21.74 ± 18.74	113.22 ± 39.51	< 0.001
Tumor kurtosis (×) value	38.5 ± 69.5	44.2 ± 61.7	0.57
Tumor max HU value	424.5 ± 374.8	550.0 ± 273.4	0.06
Tumor mean HU value	96.7 ± 23.2	79.5 ± 14.8	0.04
Tumor median HU value	97.7 ± 23.0	81.0 ± 17.3	0.06
Tumor min HU value	− 271.8 ± 290.2	− 470.0 ± 366.0	0.10
Tumor skewness (–) value	− 2.18 ± 3.74	− 3.14 ± 5.00	0.32
Standard deviation of tumor HU value	30.6 ± 6.9	34.6 ± 10.3	0.19
Tumor total HU value	1.8 × 10^7^ ± 1.9 × 10^7^	8.6 × 10^7^ ± 3.9 × 10^7^	< 0.001

### Association between SPECT/CT LSF and CECT factors

Univariate linear regression analysis revealed that the tumor volume (*P* = 0.003), total liver volume (*P* = 0.01), tumor-to-liver volume ratio (*P* < 0.001), tumor integral total (HU × L) value (*P* < 0.001), and tumor minimum HU value (*P* = 0.007) were related to the SPECT/CT LSF (Table 4).

**Table 4:** Univariate linear regression analysis with the lung shunt fraction measured by SPECT/CT as the dependent variable.

	Coefficient (β)	t	P value
Lung volume	− 1.2161	− 0.943	0.35
Tumor volume	3.4846	3.196	0.003
Total liver volume	2.7074	2.658	0.01
Normal liver volume	− 0.5659	− 0.225	0.82
Tumor-to-liver volume ratio	15.1760	4.433	< 0.001
Tumor integral total (HU × L) value	0.0637	4.415	< 0.001
Tumor kurtosis (–) value	0.0024	0.179	0.86
Tumor max (HU) value	0.0019	0.795	0.43
Tumor mean (HU) value	− 0.0551	− 1.471	0.15
Tumor median (HU) value	− 0.0512	− 1.374	0.18
Tumor min (HU) value	− 0.0069	− 2.866	0.007
Tumor skewness (–) value	− 0.4611	− 1.174	0.25
Tumor standard deviation (HU) value	0.0752	0.688	0.50

Among the significant factors from the univariate analysis, tumor volume, tumor-to-liver volume ratio, and tumor integral total (HU × L) value had a VIF higher than 10 (16.7, 14.1, and 21.7, respectively)^25^. Among these three factors, the tumor integral total (HU × L) value, which had the highest VIF value, was included in the final multivariate analysis. Multivariate analysis revealed that only a higher tumor integral total (HU × L) value was significantly correlated with a high SPECT/CT LSF value (*P* = 0.004)) (Table 5). Adjusted P values and 95% confidence intervals were obtained across the bootstrap samples.

**Table 5:** Multivariate linear regression analysis with the lung shunt fraction measured by SPECT/CT as the dependent variable.

	Multivariate regression			Bootstrap		
	Coefficient (β)	t	*P* value	VIF	*P* value	95% CI
Constant	5.4948					
Total liver volume	− 2.4654	− 1.411	0.17	3.86	0.321	− 7.532 to 3.244
Tumor integral total (HU × L) value	0.0899	3.151	0.004	3.82	0.009	0.033 to 0.170
Tumor min (HU) value	− 0.0018	− 0.575	0.57	2.19	0.636	− 0.009 to 0.005

*C.I*. confidence interval, *VIF* variance inflation factor.

**Figure 1:**
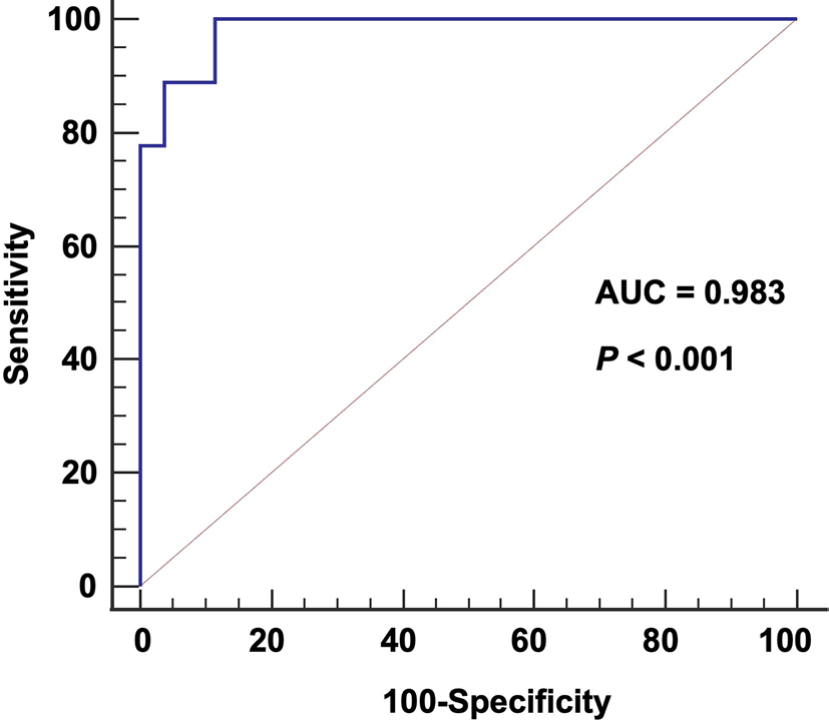
Receiver operating characteristic (ROC) curves of the integral total (HU × ml) value for distinguishing patients to receive more than 30 Gy to the lung while receiving 200 Gy to the tumor.

The tumor integral total (HU × L) value revealed a significant model generalization for predicting the patients to receive ELD > 30 Gy (Fig. 1). The area under the curve was 0.983 (95% confidence interval 0.893–1.000, bootstrap P < 0.001). The maximum Youden index was 0.88 (95% confidence interval 0.667–0.962, bootstrap), providing a sensitivity of 100% and a specificity of 88.5%.

**Figure 2:**
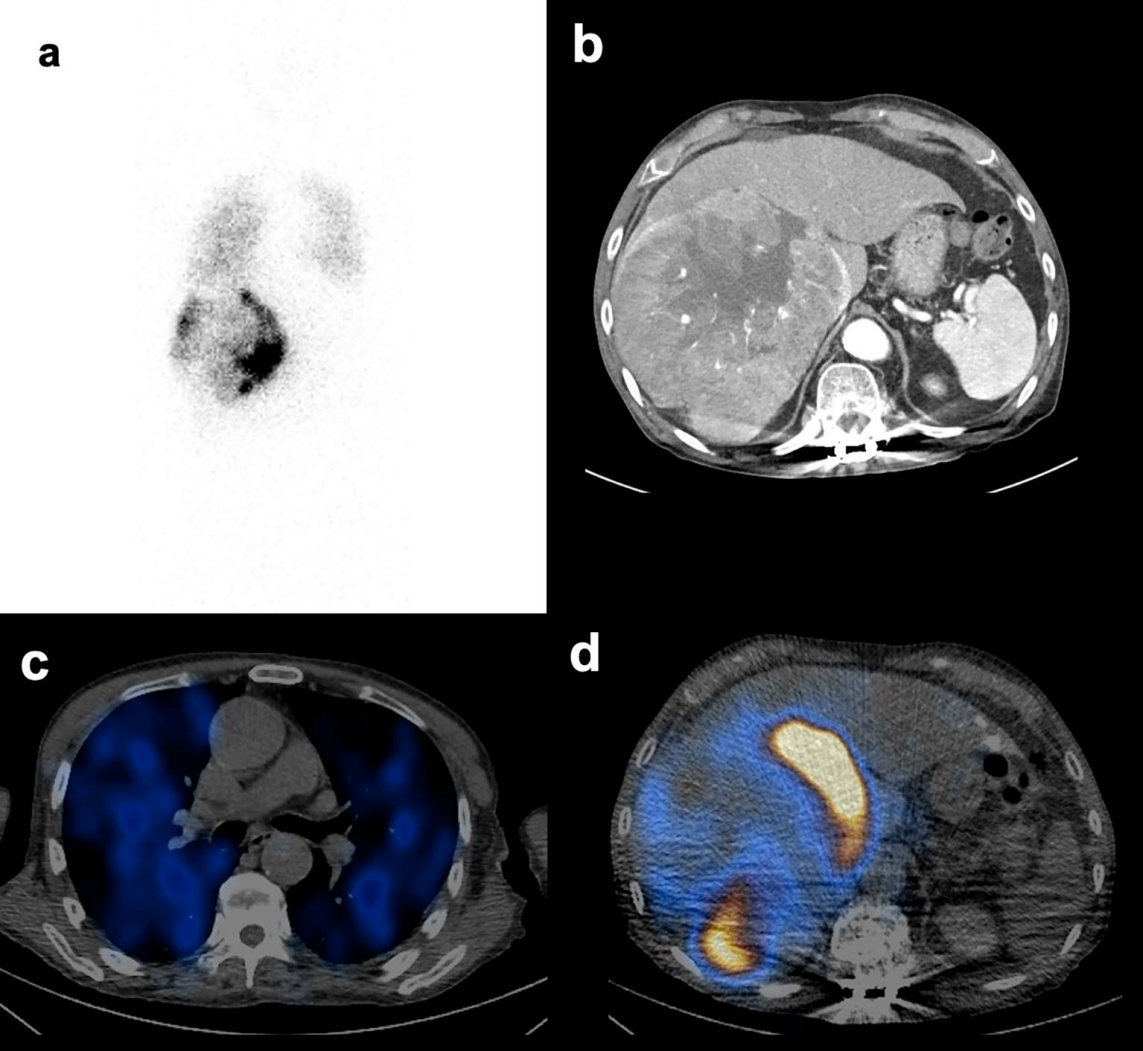
Representative case of a patient with high LSF. The patient (76-year-old male) had an LSF of 29.1% on the planar scan (**a**). He had a large sized tumor (1730 L) with central necrosis, and an integral total (HU × L) value of 141.28 (**b**). From the SPECT/CT, LSF was 14.1% (**c**), and the tumor uptake was 82.1% (**d**).

**Figure 3:**
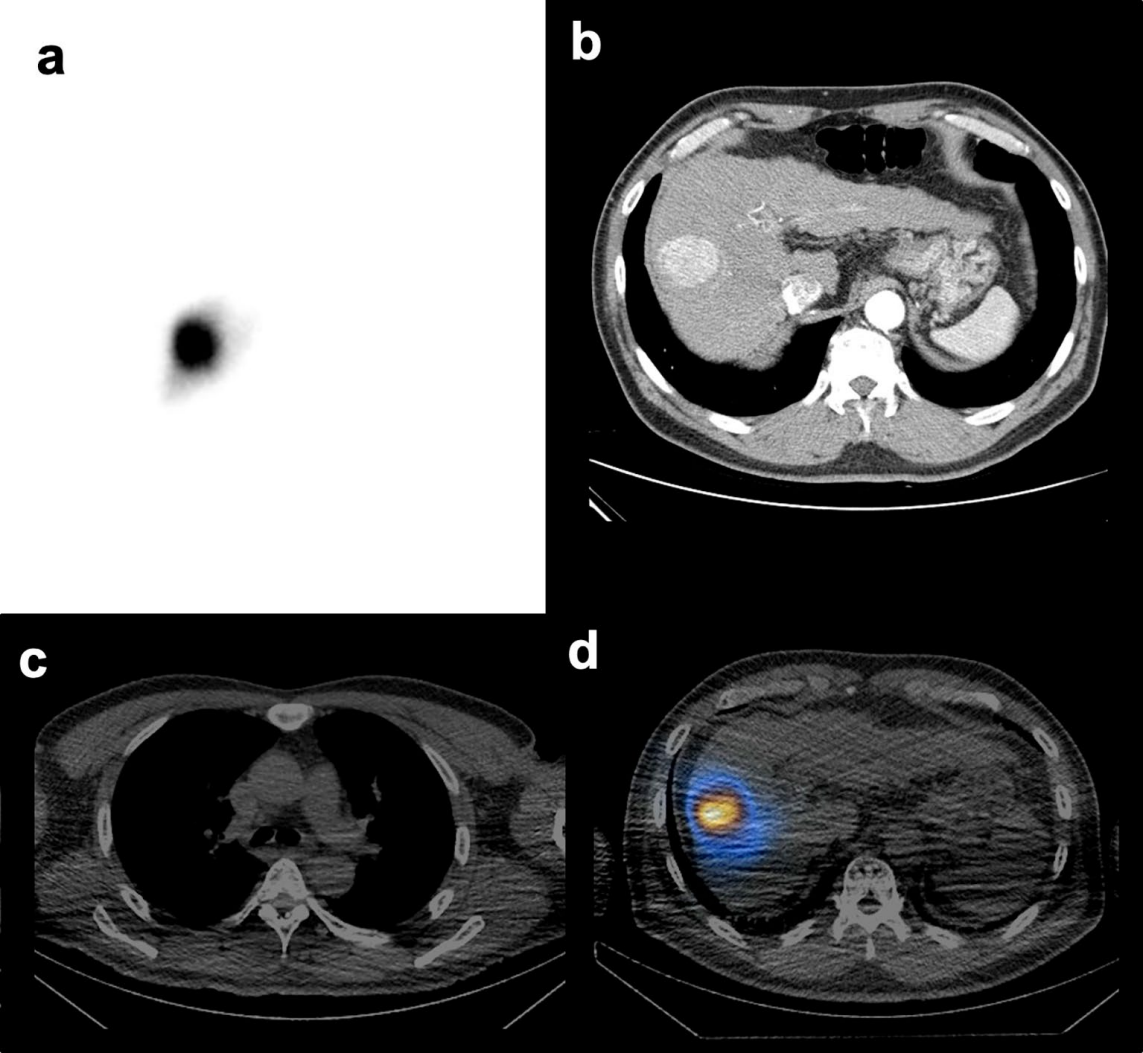
Representative cases of a patient with low LSF. The patient (58-year-old male) had an LSF of 2.7% on the planar scan (**a**), with a smaller sized tumor (36 ml), with an integral total (HU × L) value of 4.08 (**b**). From the SPECT/CT, LSF was 3.2% (**c**), and the tumor uptake was 87.5% (**d**).

Figures 2 and 3 illustrates representative cases of patients with high and low LSF.

## Discussion

This study provides pre-TARE CECT predictive factors for high LSF with quantitative evaluation using ^99m^Tc-MAA SPECT/CT. Several studies have investigated the association between CECT findings and LSF^12,13,26^. Hepatic vein invasion/thrombus or shunting^12,13^, portal vein invasion^26^, large tumors, and dysmorphic intra-tumoral vessels^12^ were identified as possible factors associated with high LSF. Our perspective on the comparison with our research is as follows. First, LSF values were measured using planar ^99m^Tc-MAA scans, whereas shunt values were calculated using the geometric mean counts of the anterior and posterior images of the lung and liver. LSF measurement using planar scans has limitations compared with LSF measurement using SPECT/CT. The overlying and underlying activity of the large tumors to the lungs cannot be excluded in planar imaging as compared to SPECT/CT^27^. Therefore, LSF values calculated using planar scans are significantly overestimated than the actual values, compared with those calculated using SPECT/CT^28^ as in our study. Furthermore, LSF value measured using SPECT/CT has advantages over planar scans because they positively correlate with the LSF measured from post-TARE ^90^Y microsphere positron emission tomography/CT^15^. Second, the LSF results of these studies were analyzed with a cut-off value of > 20% for the high-risk group, whereas in our study, we quantified the respective percentages of LSFs based on quantification with ^99m^Tc-MAA SPECT/CT scans. Third, prior studies included morphological features of the CT findings that are potentially subjective and observer-dependent, compared with quantitative parameters from texture feature analysis. To overcome these shortcomings, we analyzed LSF value by calculating values from lung perfusion SPECT/CT and correlated the LSF value with data derived from texture feature analysis.

In this study, the high tumor integral total (HU × L) value was an independent predictor for high LSF. The tumor integral total (HU × L) value reflects the size and degree of arterial enhancement, meaning that larger tumors with a higher portion of blood supply from the hepatic artery are more prone to have higher LSF. Previous studies connecting enhancement patterns and the pathological differentiation of HCC have revealed that large tumors (> 2 cm), hypervascularity, and intra-tumoral vessel/aneurysm are correlated with poor pathological differentiation^16,29^. Moreover, large or poorly differentiated tumors are associated with relatively rapid washout, suggesting abnormal drainage routes to hepatic veins^16,30^. Thus, a high tumor integral total value may indicate an increased density of abnormal vessels and arteriovenous shunts, resulting in higher LSF levels^16,31,32^. Moreover, measuring the tumor integral total (HU × L) value has the advantage of being easily accessible for estimation of LSF, as compared to other conventional methods. Additionally, it has high sensitivity and specificity for predicting the LSF. As selecting suitable patients based on precise dosimetry and LSF estimation is crucial for the safety and effectiveness of TARE, the results from this study may be useful in guiding patient selection before pre-treatment dosimetry and lowering the probability of drop-outs after pre-treatment dosimetry.

This study has some limitations. First, the number of patients enrolled was relatively small. However, the bootstrapping procedure in our study demonstrated the robustness of our model. Second, this study did not consider the differences between pre-TARE dosimetry using ^99m^Tc-MAA scans and the actual TARE dosimetry, since the purpose was to find associated parameters of the CECT with high LSF on pre-TARE dosimetry. Unlike ^90^Y microspheres, ^99m^Tc-MAA particles tend to degrade into smaller particles that distribute to other organs by passing through the capillary bed^33^. Thus, the authors attempted to reduce the time interval between ^99m^Tc-MAA injection and image acquisition to prevent unnecessary particle degradation, which could bias the LSF estimation^33^. Further studies comparing ^90^Y microsphere positron emission tomography/CT or ^90^Y bremsstrahlung SPECT/CT are required. Third, although a history of trans-arterial chemoembolization is not a contraindication for TARE, patients with a history of trans-arterial chemoembolization were excluded from this study due to the alteration of Hounsfield units on the CT image resulting from lipiodol uptake.

In conclusion, this study suggests that texture analysis of CECT scans can assist in the selection of TARE candidates with low LSF. Owing to the demerits of the two-day protocol such as treatment delay, recent protocols have suggested same-day TARE therapy with ^99m^Tc-MAA scan or even omission of pre-TARE ^99m^Tc-MAA scans^34–36^. Applying the results of our study to clinical practice may potentially help reduce the time required between diagnosis and treatment.

## Data Availability

The authors confirm that the published data in this article are available, and raw data supporting the findings could be shared by the corresponding author upon reasonable request.
